# Our Position

**Published:** 1871-03

**Authors:** 


### OUR POSITION.

As Medical Journalists, we have many things to cheer and encourage us. Our enterprise has everywhere met with friends. To all, we tender our warmest thanks. To the editorial fraternity we are brought under special obligations for the kind notices they have given the COMPANION. To the medical press, we owe a debt of gratitude for the promptness with which they have forwarded us their publications.

In this connection, we desire to state more explicitly than heretofore, some of the objects we have in view. Underlying everything, our great aim and desire is, to devote our Journal for the good, advancement and elevation, of the whole profession. There never was a time of more peril to our cause—there never was a time, in the history of medicine, when there was so great reason to apprehend subversion of the truth, and the overthrow of those principles upon which rests the honor, integrity and success of the profession, as a science. There is not a thinking physician who does not deplore the present status of his science. Hence it will be our purpose to elevate the moral and scientific standard of the profession ; to proclaim the truth, as announced in medical law, and to exhort our brethren everywhere, to harmony of sentiment, and unity of action, in its assertion and vindication.

In attempting to do this, we shall have no private interests to subserve. We do not seek to proclaim our virtues upon the house-tops, or play the roll of the Pharisee. Neither do we, of choice, desire to assume the office of censors, to condemn and anathematize medical heresies or heretics. We are not the partisans of any clique, or the apologists of any set of men. We put all under the law, as the final test of right and honor. We appeal to the law, and to those under the law. With outsiders, we or the law have nothing to do. By their action, they must stand or fall in professional or public estimation.

Taking into view the good of the entire profession, we shall not limit our vision to our own circumscribed horizon. We do not propose to dwarf our cause to one small spot of earth. The interests of one locality should command the consideration of every other. An ulcer upon one portion of the body is felt by every other part. We, therefore, shall not advocate the interests and claims of one section, and ignore the rest. We shall place our finger upon the great pulse of the profession, and to the best of our ability, shape our action for the welfare of the entire body. We hope, at all times, to have the moral courage to speak for the right—to fearlessly and boldly advocate the truth and defend the principles to which the profession is pledged. This privilege and duty we will neither barter nor sell. We will not stifle our convictions of duty or stultify our obligations to the profession, by proclaiming the value and power of medical ethics at home, and ignore them abroad. We shall not hesitate to defend our brother, wherever he may be, when assailed by professional cormorants and hyenas at home, simply because his home happens not to be ours. We will make his cause our cause—his fight our fight.

Moreover, when the law has been appealed to, and has pronounced its sentence, we shall not go behind that law to seek a pretext to disregard or to violate it. There can be no virtue in law, if, at our option, we disobey its mandates with impunity.

There can be no power in organization, if the organic law upon which it is founded, is made to subserve the interests, and sustain the errors of disorganizers. There can be no union or harmony of feeling, if law is made to yield up its claims at the suggestion of avarice, or to the behest of the lawless. With the good, the true, the noble, there is no alternative—no choice—between right and wrong; between truth and error; no happy "middle position," which can be safely occupied in the maintenance, or defiance of, law. It must be supported or denounced. It must be obeyed or derided.

As Medical Journalists, it may happen that we shall unwittingly recognize the claims of disorganizers, or men who have forfeited their claims to professional esteem. Should we ever, by accident, recognize parties who are not recognized by the proper organizations at home, when informed of the fact, the high demands of self-respect shall bring the corrective. We will neither "give sleep to our eyes, nor slumber to our eyelids" until the foul blot shall be washed from our conscience. No plea of tender-heartedness or of personal friendship shall ever seduce us from the pathway of right, or the inflexible demands of professional law.

